# Randomized Controlled Trial Comparing Primary and Staged Basilic Vein Transposition

**DOI:** 10.3389/fsurg.2015.00014

**Published:** 2015-04-29

**Authors:** Stavros K. Kakkos, Ioannis A. Tsolakis, Spyros I. Papadoulas, George C. Lampropoulos, Evangelos E. Papachristou, Nikolaos C. Christeas, Dimitrios Goumenos, Miltos K. Lazarides

**Affiliations:** ^1^Department of Vascular Surgery, University Hospital of Patras, Patras, Greece; ^2^Department of Nephrology, University Hospital of Patras, Patras, Greece; ^3^Department of Interventional Radiology, University Hospital of Patras, Patras, Greece; ^4^Department of Vascular Surgery, Democritus University Hospital, Alexandroupolis, Greece

**Keywords:** arteriovenous recommend that fistula, basilic vein, transposition, maturation, patency

## Abstract

**Objective:**

It is unclear if brachio-basilic vein fistula should be performed as a primary or staged procedure, particularly for smaller basilic veins. Our aim was to report on a randomized controlled trial comparing these two techniques.

**Methods:**

Sixteen patients with a basilic vein ≥2.5 mm were randomized into primary transposed brachio-basilic vein (TBBV) fistula (*n* = 9) and staged TBBV fistula (*n* = 7). Patients with basilic veins enlarged by previous arteriovenous fistulas were excluded. Baseline characteristics of the two study groups, including vein size, were comparable (median basilic vein diameter 3.5 mm, range 2.8–4.1 mm). The staged group had a brachio-basilic vein fistula performed first followed by the transposition procedure performed at least 6 weeks later to allow the basilic vein to enlarge. TBBV fistula maturation at 10 weeks, primary, assisted-primary, and secondary patency were the primary outcome measures. Early failures were included in the calculation of patency rates.

**Results:**

Transposed brachio-basilic vein fistula maturation rate after primary procedures (3/9, 33%) was lower compared to maturation rate after staged procedures (7/7, 100%, *P* = 0.011, Fisher’s exact test), which led to premature termination of the trial. Time to hemodialysis [median (interquartile range)] of primary and staged procedures was 54 (51.5–113.5) days and 97 (93–126) days, respectively (*P* = 0.16). One-year primary and assisted-primary patency rates of primary and staged procedures were equivalent (44 vs 57%, *P* = 0.76 and 44 vs 71%, *P* = 0.29, respectively); however, there was a trend toward a better 1-year secondary patency after staged procedures (86 vs 44% for primary procedures, *P* = 0.09).

**Conclusions:**

Among candidates for TBBV fistula with a small basilic vein, staged transposition achieves higher maturation rates compared to primary procedures, a difference reflected in long-term secondary patency.

**Trial registration:**

www.ClinicalTrials.gov, identifier NCT01274117.

## Introduction

Current guidelines recommend that transposed brachio-basilic vein (TBBV) fistulae (1) should be preferred over prosthetic grafts in patients with unsuitable cephalic veins or exhausted cephalic vein options (2), because they have better primary patency and lower infection rates and are associated with better patient survival when compared with prosthetic grafts.

The original technique of TBBV fistula was a primary basilic vein transposition, i.e., a one-stage procedure (1). More recently, staging of TBBV fistula was suggested (3), with the arteriovenous anastomosis performed during the first stage and the formal transposition performed after a period of several weeks. Interval arterialization makes staged transposition easier, because the basilic vein is enlarged and its walls are thickened and not small and friable, and perhaps faster than primary transpositions. Additionally, a small and thin-walled basilic vein can be twisted or compressed by a hematoma in the transposition tunnel or can be short, requiring a more proximal anastomosis. In case steal syndrome or venous hypertension develops after a primary TBBV fistula, an extensive and complex operation has been performed with no benefit. In staged procedures, these complications can be managed before the transposition is performed. The obvious disadvantage of procedure staging is the additional delay in TBBV fistula use.

There is some supportive evidence in favor of staged TBBV fistula from a single-center study, which found improved maturation rates with procedure staging compared with the primary technique (4). However, this study was performed in the early 1990s when preoperative vessel mapping was not used, the randomization method that was used was not reported and the exclusion criteria were not provided. At present, it remains unclear if TBBV fistula should be performed as a primary (one-stage) or staged (two-stage) procedure, particularly in smaller basilic veins. The equivalent results of case–control studies (5, 6), suggest that staging might not be necessary; however, their findings could be the result of bias, because staged procedures are usually performed in patients with basilic veins of smaller diameter.

The aim of the present study was to report on a randomized controlled trial (RCT, ClinicalTrials.gov identifier, NCT01274117) comparing the short- and long-term results of primary and staged TBBV fistula.

## Materials and Methods

Suitable patients with chronic kidney disease (CKD) were randomized to have a TBBV fistula performed either as a primary or a staged procedure (randomization ratio 1:1). The protocol appears on www.clinicaltrials.gov (identifier NCT01274117).

### Inclusion and exclusion criteria

Patients between 18 and 90 years of age of both genders, with CKD already on hemodialysis or with anticipated hemodialysis were eligible for inclusion. Patients were excluded if they were unwilling to participate and/or not consenting, had a suitable cephalic vein to construct a radio-cephalic or brachio-cephalic fistula, the basilic vein was unsuitable for use because it measured less than 2.5 mm or had intrinsic lesions on color-coded Duplex ultrasound (CCDU), or the basilic vein was already enlarged by a previous wrist or elbow arteriovenous fistula draining into the basilic vein on CCDU, as detailed below. Preoperative vessel mapping was performed with CCDU and selective use of venography (7), with the minimum and maximum diameter of the basilic vein being recorded, after application of a tourniquet, including the diameter of the medial antecubital (basilic) vein. Particular attention was made to identify drainage patterns of previous AVFs (radio-cephalic and brachio-median cubital) leading to enlargement of the basilic vein in order to exclude these patients from the trial and also to completely map the basilic vein in order to choose the incision site (8).

Patients were recruited from the Vascular Access Outpatient Clinic of the University Hospital of Patras, Greece, and enrolled during the period between December 2010 and April 2013. The study protocol was approved by the Ethics Committee of the University Hospital of Patras, Greece; all participants provided written informed consent and the trial conformed to the Declaration of Helsinki.

### Surgical techniques

All procedures were performed by the first author using magnifying loupes (×2.5) under local anesthesia with lidocaine 1% (Xylocaine, AstraZeneka, Södertälje, Sweden), preferentially on an outpatient setting. Heparinization was performed with 2,500 iu of heparin sodium in the form of basilic vein flush.

Primary procedures (1, 5, 9) were performed with the modification of skip incisions to dissect the basilic vein. A large subcutaneous tunnel was created (10), and a 7-mm brachial arteriotomy was used. The staged group had a brachio-basilic vein (BBV) fistula performed first, through a transverse antecubital incision approximately 1 cm distal to the elbow crease using the same arteriotomy length (7 mm) as in primary procedures. Whenever the median antebrachial vein was not suitable, the main basilic vein was used to create the anastomosis through a longitudinal incision. The transposition procedure was performed at least 6 weeks later to allow the basilic vein to enlarge, guided by an interval CCDU to confirm this (desirable vein diameter ≥6 mm), using skip incisions, basilic vein transection, placement into the tunnel, and reanastomosis (5). Previously described modified techniques of basilic vein transposition, guided by the preoperative CCDU and operative findings were selectively used when indicated (11–13).

### Outcome measures

Primary outcomes included TBBV fistula maturation defined as usage of the fistula for three consecutive sessions with two needles (or clearance for use in case of pre-dialysis) between 6 and 10 weeks after the transposition procedure (main primary outcome measure subject to power calculations), and long-term primary, primary assisted, and secondary patency.

Reporting standards for arteriovenous accesses of the Society for Vascular Surgery and the American Association for Vascular Surgery were used to define access patency (14). Primary patency was defined as fully functional access, the first episode of failure or thrombosis being used to determine this outcome. Assisted-primary patency was defined as patent access without thrombectomy, including those in which any intervention had been performed to prevent what was perceived to be impending failure, so that patency is lost when the first thrombosis occurred. Secondary patency was defined as accesses that were patent after thrombectomy, the terminal thrombotic event being taken into account to estimate secondary access patency rates.

Secondary outcome measures included basilic vein diameter on ultrasound at 4 weeks after the procedure, overall maturation (irrespective of time required to achieve this), postoperative and long-term complications (including hematoma, steal syndrome, and venous hypertension), and full time to usage of the TBBF fistula (or clearance in case of pre-dialysis) after the initial procedure.

### Sample size determination

Based on the results of a similar study (4), where maturation rates of 90% for staged procedures and 60% for primary procedures were reported, it was estimated that 20 patients in each group would be required for the main primary outcome measure, maturation (α = 0.05, β = 0.10). A formal interim analysis was not planned; however, maturation results were closely monitored because of the open-label design of this surgical trial.

### Randomization

All patients provided written informed consent before entering the study. Randomization was performed using sequentially numbered sealed opaque envelopes, stratified by vein size (2.5–3.9 mm and ≥4 mm, based on previously suggested empirical evidence) (8). To avoid crossover of patients and minimize the possibility of consent withdrawal, the envelopes were opened up at the time of surgery, before the incision was made. Obviously, blinding was not possible for this trial on surgical procedures; however, with the course of time, the antecubital fossa scar tended to fade-away reducing detection bias, which was nevertheless based on objective hard endpoints of maturation and patency.

### Statistics

All data of our RCT were entered into a Microsoft Office Access database (Microsoft Inc., Redmond, WA, USA) and analyzed with IBM SPSS Statistics 21 (IBM Corp., Armonk, NY, USA). All access patency rates were calculated with the Kaplan–Meier method and compared with the log-rank (Mantel–Cox) test. Patients were followed-up for a minimum of 1 year and up to the point secondary patency was lost, while initial failures were included in the calculation of long-term patency rates. Categorical data were analyzed with the chi-square or Fisher’s exact test, where appropriate; numerical data were analyzed with the Mann–Whitney test, where appropriate. Relative risk for non-thrombotic complications was calculated, but this was not estimable for maturation because of the absence of events in the staged TTBV fistula group. Similarly, relative risks for long-term patency results were calculated using Cox proportional hazards modeling. A *P*-value <0.05 was considered as statistically significant. Two-sided statistical tests were always used.

## Results

**Figure 1:**
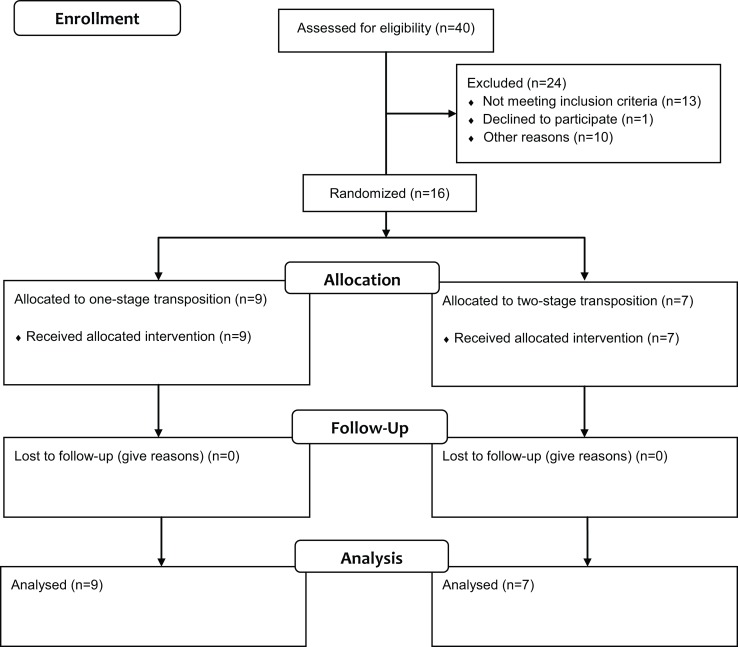
**Consolidated Standards of Reporting Trials (CONSORT) Statement 2010 flow diagram showing the enrollment, allocation, follow-up, and analysis stages of our RCT**.

Forty patients were assessed for eligibility, with 24 of them being excluded as shown in Figure 1. Of those excluded, 13 patients did not meet the inclusion criteria and were not randomized: six patients had enlarged basilic veins – median diameter 7.1 mm and range 4.5–9.2 mm – from previous fistulas and underwent a primary TBBV fistula; seven patients had anatomical reasons, including small basilic vein in five of them, small brachial artery in one of them, and extensive scarring at the elbow due to previous brachio-cephalic fistula, all but one undergoing placement of a prosthetic graft; one patient refused to participate and an additional 10 patients were excluded, per investigators’ choice to receive a loop forearm graft (*n* = 2) at the beginning of our series or an upper arm graft (*n* = 7), or for other reasons (*n* = 1).

The remaining 16 patients were randomized into primary TBBV fistula (*n* = 9) and staged TBBV fistula (*n* = 7). Baseline characteristics (demographics, pertinent history and co-morbidities) of the two study groups are demonstrated in Table 1 and were comparable; however, a marginally significant difference for antithrombotic treatment being used more frequently in two-staged procedures was noted. Only four patients had a basilic vein of 4 mm or larger and three of them were randomized to primary TBBV fistula. Similarly, preoperative vessel mapping results, including minimum basilic vein size, were comparable (Table 2). The median minimum basilic vein diameter of both groups was 3.5 mm (range 2.8–4.1 mm). A relevant finding in primary procedures was one case of a duplicated basilic vein, while in four patients of staged procedures, there were quality issues of the medial basilic vein (*n* = 3) or scarring due to a previous brachio-cephalic fistula (*n* = 1) requiring the main basilic vein to be anastomosed to the brachial artery (or the radial artery in a patient with a known high bifurcation) at the level or just above the elbow crease.

**Table 1:** **Demographics, pertinent history and co-morbidities of patients randomized into the two study groups, primary and staged TBBV fistula**.

	TBBV fistula		*P* value	All patients
	Primary (*n*=9)	Staged (*n*=7)	
Age [median, (IQR), years]	61 (53.5–71)	59 (43–67)	0.68	60.5(50.5–67.0)
Gender (male/female)	3/6	4/3	0.62	7/9
Race (caucasian/other)	9/0	6/1	0.44	15/1
Pre-dialysis (%)	0 (0%)	1 (14.3%)	0.44	1 (6.3%)
Chronic kidney disease cause (%)
Diabetes mellitus	2 (22.2%)	0 (0%)	0.32	2 (12.5%)
Hypertension	1 (11.1%)	1 (14.3%)		2 (12.5%)
Glomerulonephritis	3 (33.3%)	3 (42.9%)		6 (37.5%)
Interstitial disease	1 (11.1%)	1 (14.3%)		2 (12.5%)
Cystic disease	0 (0%)	2 (28.6%)		2 (12.5%)
Unknown	2 (22.2%)	0 (0%)		2 (12.5%)
History of previous ipsilateral access (%)	4 (44%)	4 (57%)	1.00	8 (50%)
Radio-cephalic AVF	2 (22%)^a^	3 (43%)^b^	0.60	5 (31.3%)
Brachio-cephalic AVF	2 (22%)^b^	1 (14%)^b^	1.00	3 (18.8%)
Co-morbidities	7 (78%)	7 (100%)	0.48	14 (87.5%)
Diabetes mellitus	1 (11%)	2 (28%)	0.55	3 (18.8%)
Hypertension	3 (33%)	4 (57%)	0.62	7 (43.8%)
Other	6 (67%)	6 (86%)	0.59	12 (75%)
Antithrombotic treatment	0 (0%)	3 (43%)^c^	0.06	3 (18.8%)

*AVF, arteriovenous fistula*.

*^a^All failed to mature*.

*^b^One AVF failed to mature, two AVFs used for 18 and 24 months, respectively*.

*^c^Two patients on antiplatelets and one on anticoagulation with acenocoumarol*.

**Table 2:** **Ultrasound findings and procedure details of patients randomized into the two study groups, primary and staged TBBV fistula**.

	TBBV fistula		*P* value
	One-stage (*n*=9)	Two-stage (*n*=7)	
Preoperative basilic vein diameter [median, (IQR), mm]
Minimum	3.7 (3.2–4.0)	3.3 (3.2–3.4)	0.30
Maximum	5.5 (4.8–6.3)	5.1 (3.3–5.7)	0.41
Preoperative brachial artery diameter [median, (IQR), mm]	4.5 (3.8–4.7)	4.5 (3.7–5.6)	0.76
TBBV fistula side (L/R)	6/3	3/4	0.62
Duration of first procedure^a^ [median, (IQR), min]	220 (163–270)	115 (75–120)	<0.001
Postoperative complications of first procedure^a^	5/9 (56%)	2/7 (29%)	0.36^b^
30-day basilic vein diameter [median, (IQR), mm]
Minimum	5.9 (4.7–6.9)	4.6 (3.5–5.2)	0.09
Maximum	7.3 (6.2–7.8)	7.8 (6.7–8.5)	0.28
Time interval between stages in 2-stage procedures [median, (IQR), min]	N/A	55 (47–76)	N/A
Duration of transposition in 2-stage procedures [median, (IQR), min]	N/A	180 (180–205)	N/A
Postoperative complications of transposition in 2-stage procedures	N/A	2/7 (29%)	N/A
Postoperative complications (all stages)	5/9 (56%)	3/7 (43%)	1.00^c^

*^a^TBBV fistula and BB fistula, respectively*.

*^b^Relative risk 1.75, 95% CI 0.78–3.93*.

*^c^Relative risk 1.29, 95% CI 0.49–3.40*.

Basilic veins were equally enlarged 1 month after the TBBV fistula (primary procedures) or the BBV fistula (staged procedures), with median minimum fistula diameter being 5.9 vs 4.6 mm, respectively, *P* = 0.09 (Table 2). The median (interquartile range) interval between the stages of the staged group was 55 (47–76) days. In four of the patients undergoing the second stage of the staged procedure, a technical modification was required in the form of concomitant brachial vein harvesting (because of a low basilic vein junction), relocation of the brachial anastomosis above the elbow crease (because of small – 3 mm – diameter of the proximal basilic vein, apparently a failure to enlarge), resection of a proximal fibrotic/stenotic part and creation of the anastomosis with the two fistula stumps, and division of the basilic vein near the axilla and reanastomosis (because of a 5 mm/partially enlarged proximal basilic vein, likely to fail or become stenotic if anastomosis was to be made at this level). One more patient with a duplicated basilic vein underwent transposition of both ramii, each measuring at least 6 mm in diameter.

TBBF fistula maturation by week 10 with staged operations (7/7, 100%) was significantly better compared to primary operations (3/9, 33%, *P* = 0.011, Fisher’s exact test), but also final maturation rate with staged operations (7/7, 100%) was significantly better compared to primary operations (4/9, 44%, *P* = 0.034, Fisher’s exact test), which led to premature termination of the trial. More specifically, one TBBV fistula was found thrombosed by the 1-month follow-up, a second one was thrombosed while the patient was awaiting repair of an anastomotic pseudoaneurysm (see below), a third one was abandoned due to low flows and soon was thrombosed, a fourth one thrombosed during the second postoperative month before it was deemed mature, and the fifth one was thrombosed before fully used (one needle trial) possibly in relation to prolonged bleeding requiring application of local pressure. The single patient on pre-dialysis started using his TBBV fistula 1 year after it was released for use. There was no association between TBBF fistula maturation by week 10 and final maturation rates and use of antithrombotics (*P* = 0.25 and *P* = 0.51, respectively).

Time to hemodialysis was longer after staged procedures compared to primary procedures (Table 3). Non-thrombotic postoperative and TBBV fistula-related complications after primary and staged procedures occurred in 5/9 (56%) and 3/7 (43%) patients, respectively (*P* = 1.00, Fisher’s exact test, Table 2, RR 1.29, 95% CI 0.49–3.40). In the primary TBBV fistula group, these complications included extensive arm bruising and hematoma (requiring hospitalization most likely due to an anastomotic pseudoaneurysm diagnosed at 30 days) in two patients, respectively, a puncture-related pseudoaneurysm (requiring operative repair) in a third patient, prolonged bleeding in a forth patient and re-circulation due to the short length of the available basilic vein (requiring complementary transposition) in the fifth patient. In the staged group, these complications included grade 1 steal syndrome (after the BB fistula, spontaneously resolved), grade 1 venous hypertension (after the transposition procedure, managed conservatively) and wound infection with dehiscence after both procedure stages, requiring debridement of the axillary wound and healing by third intention after the transposition in a patient with SLE on corticosteroids.

**Table 3:** **Dialysis-related outcomes of patients randomized into the two study groups, primary and staged TBBV fistula**.

	TBBV fistula		*P* value	Relative risk (95% CI)
	One-stage (*n*=9)	Two-stage (*n*=7)	
Time to hemodialysis after the first procedure [median, (IQR), days]^a^	54(51.5–113.5)	97(93–126)	0.16	–
Time to hemodialysis after the transposition procedure [median, (IQR), days]	54(51.5–113.5)	50(38–58)	0.16	–
TBBV fistula maturation by week 10	3/9 (33%)	7/7 (100%)	0.01	N/A
TBBV fistula maturation	4/9 (44%)	7/7 (100%)	0.03	N/A
Primary patency			0.76	1.23 (0.33–4.61)
Year 1	44%	57%		
Year 2	44%	43%		
Assisted-primary patency			0.29	3.38 (0.46–12.33)
Year 1	44%	71%		
Year 2	44%	71%		
Secondary patency			0.09	5.32 (0.62–45.76)
Year 1	44%	86%		
Year 2	44%	86%		

*^a^TBBV fistula and BB fistula, respectively*.

**Figure 2:**
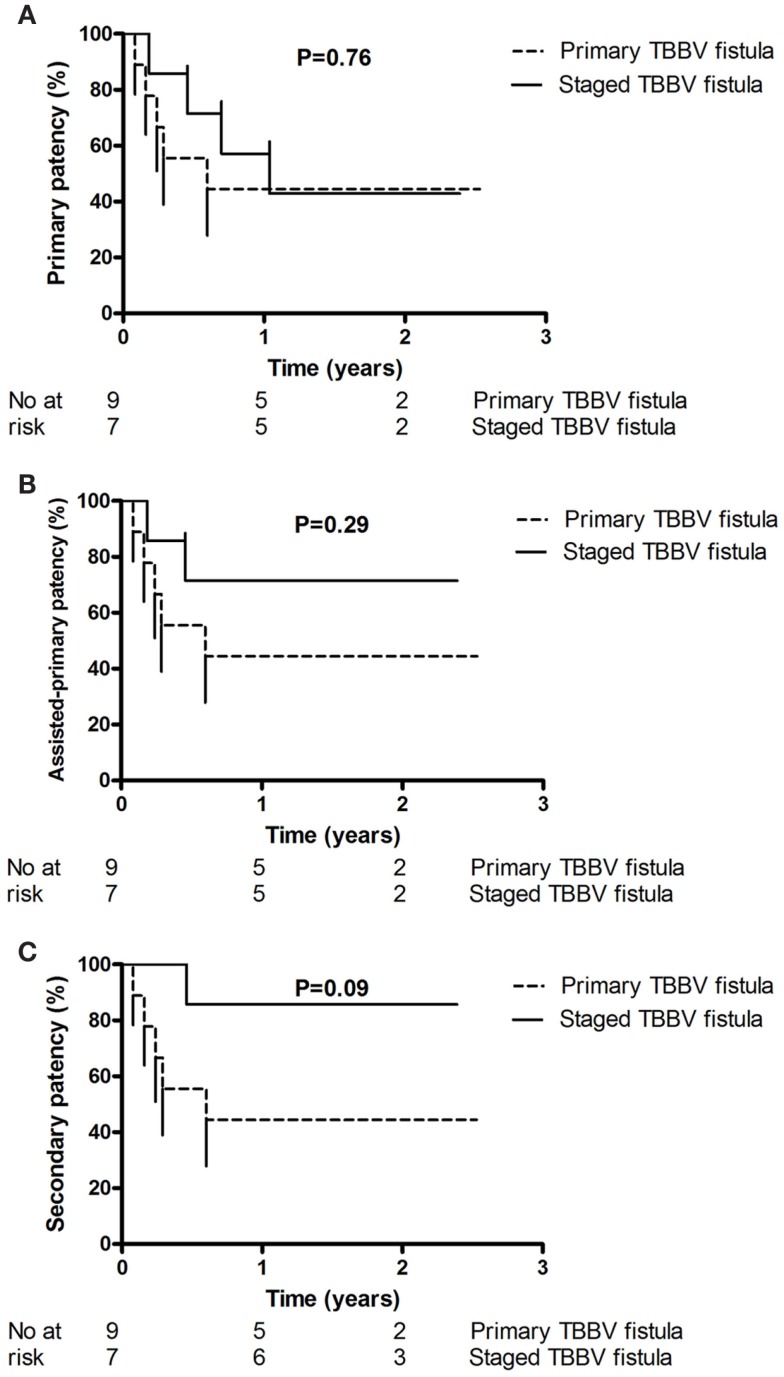
**This figure shows the primary patency (A), assisted-primary patency (B), and secondary patency (C) of the patients randomized into primary and staged TBBV fistula**. One-year primary and assisted-primary patency rates of primary and staged procedures were equivalent [44 vs 57%, *P* = 0.76, **(A)**, and 44 vs 71%, *P* = 0.29, **(B)**, respectively]. There was a trend toward a better 1-year secondary patency after staged procedures compared to primary procedures [86 vs 44%, respectively, *P* = 0.09, **(C)**].

One-year primary and assisted-primary patency rates of primary and staged procedures were equivalent (44 vs 57%, *P* = 0.76, Figure 2A, and 44 vs 71%, *P* = 0.29, Figure 2B, respectively). There was a major trend toward a better 1-year secondary patency after staged procedures compared to primary procedures (86 vs 44%, respectively, *P* = 0.09, Figure 2C); however, after the initial failures of primary procedures, the two techniques seem to perform equally well (Figure 2C). Repeat analysis excluding initial failures showed a trend for better 1-year primary patency rates of primary procedures (100 vs 57%, for staged procedures, *P* = 0.08); however, 1-year assisted-primary and secondary patency rates of primary and staged procedures were equivalent (100 vs 71%, *P* = 0.27, and 100 vs 86%, *P* = 0.45, respectively). No patient was lost to follow-up. Minimum and maximum follow-up was 1 and 3 years, respectively. All patients were alive at the end of the study except one with a well-functioning primary TBBV fistula who died 23 months postoperatively due to sepsis after heart surgery.

## Discussion

We have demonstrated that TBBF fistula maturation with staged operations is significantly better than primary procedures. Primary and assisted-primary patency of the two techniques is equivalent; however, there is a trend toward a better 1-year secondary patency after staged procedures, as a result of the worse performance of primary transposition.

The present RCT has demonstrated that maturation rates after primary procedures are significantly worse than those of staged procedures. In the absence of previous arterialization, the basilic vein can be very thin-walled and friable, susceptible to damage in primary procedures. Also, the basilic vein may be twisted or compressed by hematoma in the tunnel more often in primary procedures than in staged procedures (15). Our results, were not only expected but also in agreement with one of the two RCTs on this topic, which reported maturation rates of 90% with staged procedures vs 60% with primary procedures (4), raising the level of evidence (from level B to level A – two RCTs with consistent results) (16). We attribute our somewhat worse maturation rates with primary procedures to the fact that we randomized only relatively small basilic veins and excluded those that were arterialized based on history of proximal access and also findings of CCDU scanning. The last modality was not reported to be used in the above mentioned RCT (4), performed in the early 1990s before preoperative vessel mapping was described leading to improved results of AVF construction (17). On the other hand, the second RCT failed to demonstrate a significant benefit with staged procedures. Exclusion and inclusion criteria of the two studies were not provided. In agreement with clinical experience, basilic veins already enlarged by previous more distal AVFs are associated with good results regardless of staging or not the procedure, which could explain the difference among the three trials in addition to any bias.

In the present RCT, a delay to hemodialysis was inevitable for patients randomized into staged procedures. Given the importance of achieving the goal of TBBV fistula maturation, it seems obvious that with staged procedures the large outcome advantage outweighs the relatively short inherent delay and its attendant risks, taking into account a 10% 50-day infection rate of a hemodialysis catheter (18), and a 10% 3-year infection rate of a prosthetic graft (19), should such an option be opted. Delay to hemodialysis, an obvious disadvantage of staged procedures (5), was kept at a minimum in this RCT. Obviously, arterialized basilic veins that are usually much larger do not need a staged procedure, to avoid the risks of prolonged hemodialysis catheter use. To prevent this potential problem, others have suggested selective use of staged procedures if the diameter of the basilic vein is smaller than 4 mm, a predictor for failure of maturation (8, 20). Of note, two of the three TBBV fistulas that had a primary transposition of a basilic vein diameter ≥4 mm failed to mature; therefore, we would suggest that for a non-enlarged basilic vein a cut-off point of 5 mm to be used. Further research could be performed to prove this suggestion.

Long-term primary patency rates of the two study groups are comparable, but our study was prematurely stopped before the trend for secondary patency in favor of staged procedures became significant. Better long-term results with staged procedures compared to primary ones (RR 3.2) have been reported in a case–control study although the maturation rates in the two study groups were equivalent (6). Primary patency of the two groups of our RCT is almost indistinguishable, indicative that regardless of procedure staging, problems relating to stenotic vein segments might arise during follow-up; however, if these develop in primary transposition, they are usually irreversible because of thrombosis of a small basilic vein that is placed inside a hostile and actively remodeling subcutaneous tunnel, while in staged procedures the basilic vein is already enlarged and less likely to thrombose. Based on our RCT presented herein, it seems that after the initial failures of primary procedures the two techniques perform equally well, similarly to what has been shown by another RCT (4). The very small number of interventions that is necessary to maintain patency in our series, unlike prosthetic grafts, confirms the value of TBBV fistulas (9, 21). Postoperative and long-term non-thrombotic complications, known to occur frequently (22, 23), are in favor of staged TBBV fistulas as previously described (5), although the difference is not significant.

Meta-analyses comparing primary and staged BBV fistulas are scarce. In such a study, which included mainly case-controlled studies and only one RCT, there was a suggestion that the two-stage technique was equally good with primary BBV fistulas in terms of similar maturation and patency results (24).

The limitations of our RCT include its small size; however, its power is calculated to be 0.89 (two-sided test). An additional limitation is the fact that the study was prematurely stopped and as a result it could not detect any difference in long-term patency. The large benefit with staged procedures in patients with small and not arterialized veins precludes to our view any future trials; obviously, patients with bigger and non-arterialized veins, rare to recruit though, could be randomized into primary and staged TBBV fistula, in view of the recently reported long-term patency with the latter approach (6). Future work to our opinion should also focus on techniques to improve further staged procedures (11), including the direct comparison of superficialization with formal transposition.

In conclusion, among candidates for TBBV fistula and small basilic veins, our small study demonstrated that staged transposition is superior to primary procedures in terms of maturation, a difference persisting in the long-term secondary patency of the two groups.

## Conflict of Interest Statement

The authors declare that the research was conducted in the absence of any commercial or financial relationships that could be construed as a potential conflict of interest.
